# Microtubule plus-ends within a mitotic cell are ‘moving platforms’ with anchoring, signalling and force-coupling roles

**DOI:** 10.1098/rsob.120132

**Published:** 2012-11

**Authors:** Naoka Tamura, Viji M. Draviam

**Affiliations:** Department of Genetics, University of Cambridge, Downing Site, Downing Street, Cambridge CB2 3EH, UK

**Keywords:** microtubule plus-end, mitosis, plus-tip complexes, kinetochore

## Abstract

The microtubule polymer grows and shrinks predominantly from one of its ends called the ‘plus-end’. Plus-end regulation during interphase is well understood. However, mitotic regulation of plus-ends is only beginning to be understood in mammalian cells. During mitosis, the plus-ends are tethered to specialized microtubule capture sites. At these sites, plus-end-binding proteins are loaded and unloaded in a regulated fashion. Proper tethering of plus-ends to specialized sites is important so that the microtubule is able to translate its growth and shrinkage into pushing and pulling forces that move bulky subcellular structures. We discuss recent advances on how mitotic plus-ends are tethered to distinct subcellular sites and how plus-end-bound proteins can modulate the forces that move subcellular structures. Using end binding 1 (EB1) as a prototype plus-end-binding protein, we highlight the complex network of plus-end-binding proteins and their regulation through phosphorylation. Finally, we develop a speculative ‘moving platform’ model that illustrates the plus-end's role in distinguishing correct versus incorrect microtubule interactions.

## 2. Background and scope of the review

### 2.1. Microtubule structure and regulation

Microtubules are composed of dimers of α- and β-tubulin subunits that together generate long hollow filaments (reviewed in recent studies [1,2]). Differences in the rates of new subunit addition and removal cause a switch between the growing and shrinking states of the tubulin polymer, leading to an intrinsically dynamic polymer. The polymer has two ends: the plus- and minus-end. The plus-end is the most dynamic, and the focus of this review.

The intrinsic dynamic behaviour of microtubules is further regulated by several microtubule-associated proteins (MAPs) and motor proteins (reviewed by Akhmanova & Steinmetz [3]). Of these, a family of evolutionarily conserved MAPs and motors accumulate more at the microtubule plus-end (reviewed by Wu *et al*. [4]) compared with microtubule wall; these are termed plus-end-tracking proteins (+TIPs) [5]. We review mitotic +TIP regulation and function, by drawing a few contrasts to their interphase counterparts.

### 2.2. Mitosis versus interphase microtubules

During interphase, microtubules nucleate primarily from a single microtubule organizing centre (MTOC). At the onset of mitosis, the interphase network of microtubules undergoes sudden disassembly, allowing the rapid reassembly of a complex bipolar spindle. Concomitantly, the duplicated centrosomes separate, forming two opposing MTOCs at the spindle poles. Regional gradients of signals act in the spatial range of micrometres and promote microtubule growth towards chromosomes. By contrast, site-specific signals act in the range of submicrometres and selectively stabilize microtubules that are properly tethered to chromosomes at specialized sites, called kinetochores.

**Figure 1.**
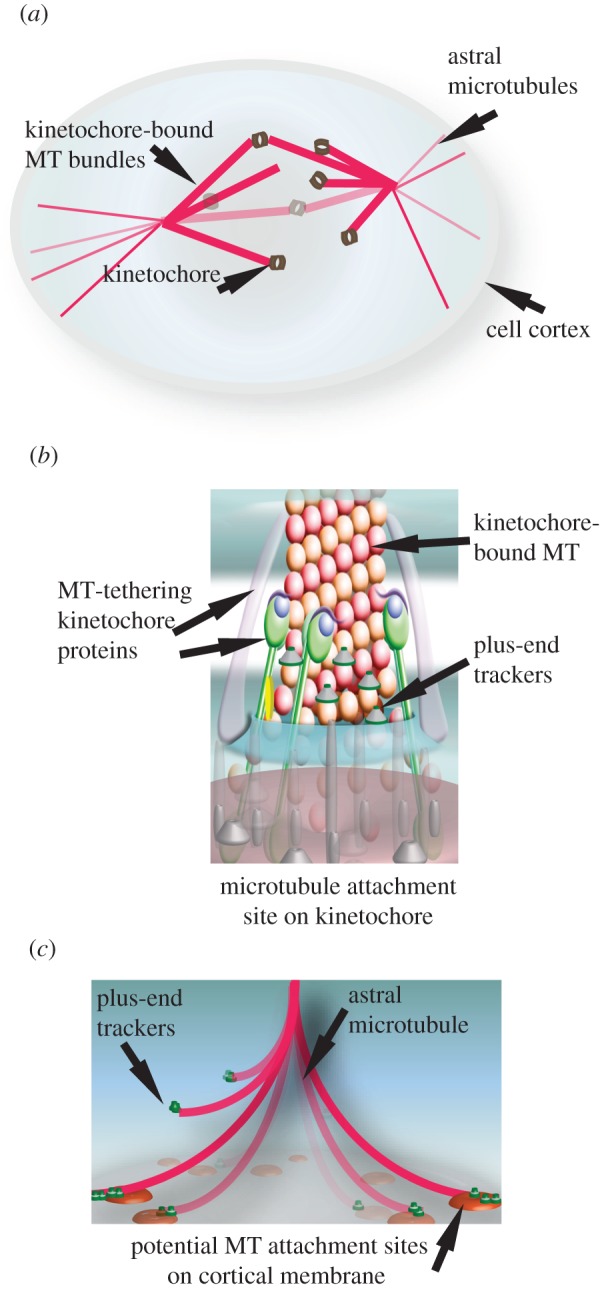
(*a*) Illustration of microtubules of the spindle apparatus with microtubule plus-ends attached to distinct subcellular sites: the kinetochore (*b*) and the cell cortex (*c*). (*b*) Illustration of the centromeric-DNA-bound multi-protein structure, the kinetochore that is tethered to microtubule plus-ends. (*c*) Illustration of the mitotic cell cortex-bound potential microtubule attachment sites for capturing and retaining microtubule ends.

Spindle microtubules are classified into three groups, on the basis of the position of the plus-end within the cell: (i) astral microtubules with their plus-ends probing the cortex, (ii) kinetochore fibres with their plus-ends tethered to chromosomes, and (iii) interpolar microtubules that emanate from one spindle pole into the central spindle towards the other spindle pole (figure 1*a;* reviewed by Compton [6]).

The rate of microtubule turnover increases tenfold in mitosis, compared with interphase. This steep increase in turnover allows the diverse roles of a mitotic plus-end, such as the rapid capture of chromosomes, pulling apart of sister chromatids and steering of the mitotic spindle towards a predetermined axis. We speculate that the plus-end bound to +TIPs acts as a ‘mobile platform’ with signalling, tethering and force-coupling roles during mitosis. To develop this model, we discuss +TIPs required for chromosome segregation and spindle positioning in mammalian cells.

## 3. Mitotic roles of microtubule plus-ends at distinct subcellular sites

Global growth and shrinkage rates of plus-ends (referred here as plus-end dynamics) determine the length of spindle microtubules; microtubule length can in turn dictate spindle shape and size [7]. At distinct plus-end interaction sites, detailed below, plus-end dynamics are differentially controlled, depending on the needs of chromosome segregation or spindle positioning events.

### 3.1. Plus-end interactions at chromosomes

Plus-ends are tethered to chromosomes at specialized submicrometre-sized macromolecular structures called kinetochores, which assemble specifically on the centromeric region of DNA. Each chromosome assembles a pair of kinetochores. Kinetochores recruit microtubule-tethering proteins and proteins that directly bind to microtubule ends (figure 1*b*). Initially, kinetochores attach to walls of microtubules (lateral attachment); subsequently, they convert the attachment to plus-ends of microtubules (end-on attachment). Lateral to end-on conversion is important because only after the establishment of an end-on attachment are the growth and shrinkage of kinetochore bound plus-ends translated into forces that push and pull chromosomes (R. Shrestha & V. M. Dravian 2012, unpublished data).

Kinetochore–microtubule attachments are stabilized when microtubule-mediated forces from opposing spindle poles pull the kinetochore pair apart. Such forces arise only when sister kinetochores are attached to microtubule ends from opposing spindle poles, a state called bi-orientation. Bi-orientation, or the correctness of kinetochore–microtubule attachment, is monitored and signalled to prevent the initiation of anaphase in the presence of an erroneous attachment. In addition to its role in ensuring bi-orientation, microtubule-mediated pulling forces are required for the accurate segregation of sister chromatids. Particularly, merotelic kinetochore pairs, where one of the sister kinetochores is bound erroneously to microtubules from both spindle poles, critically rely on anaphase pulling forces for accurate segregation [8]. A long-standing question in this area of research has been to understand how kinetochores remain tethered to plus-ends, despite the dynamic addition and removal of tubulin subunits, and resist the forces that separate chromosomes (reviewed by Cheeseman & Desai [9]).

### 3.2. Plus-end interactions at the cell cortex

Plus-ends interact with the mitotic cell cortex that recruits force generators to pull at astral microtubules and steer the spindle. It is not known whether specialized microtubule anchoring complexes assemble at the mitotic cell cortex (equivalent of lipid rafts or cell adhesion sites) to set up macromolecular platforms for capturing and establishing plus-end interaction with the cell cortex (figure 1*c*).

Plus-end interaction at the cortex is thought to guide spindle movements towards a predetermined position. In multi-cellular models, membrane-bound polarized protein complexes that define the final position of the spindle can also regulate plus-end interaction at the actin-rich mitotic cortex [10–12]. On the other hand, in non-polarized single-cell models, the retraction fibres formed at the site of cell–substrate adhesion are sufficient for plus-end interaction at the cortex and proper positioning of the spindle [13,14]. These show the presence of both polarity-dependent and -independent mechanisms to control spindle movements. The precise biochemical nature of signals that regulate plus-end dynamics at the mitotic cell cortex remains unclear.

## 4. Regulatory components of the plus-end

Plus-end-tracking proteins (+TIPs) are a wide range of MAPs and motors. They all share a common denominator of residing more at the microtubule plus-ends compared with microtubule walls. +TIP localization at plus-ends in interphase is specific to the microtubule growth/shrinkage phase (reviewed by Akhmanova & Steinmetz [3]). During mitosis, some of the +TIPs are recruited to specialized microtubule interaction sites (table 1).

**Table 1.** List of mammalian +TIPs: selected list of mammalian plus-end binding proteins to illustrate their diverse and dynamic localization through the cell cycle. ✓ and ✗ refer to ‘yes’ and ‘no’, respectively.

proteins	plus end localization		other known localization in mitosis	EB1 interactor	references
	interphase	mitosis			
APC	✓	✓	centrosome and kinetochore	✓	[15–19]
CDK5RAP2	✓	✗	centrosome	✓	[20–22]
chTOG1	✓	unclear	centrosome, cleavage furrow and spindle	not reported	[23–25]
CLASPs	✓	✓	kinetochore, central spindle and midbody	✓	[26–29]
CLIPs	✓	✓	kinetochore	✓	[30–33]
DDA3	✓	unclear	kinetochore and spindles	✓	[34–36]
Diaphanous (Dia1)	✓	unclear	spindles	✓	[37,38]
Dynactin (p150)	✓	✓	centrosome, cortex, spindle and kinetochore	✓	[39–44]
Dynein	✓	✓	centrosome, cortex, spindle and kinetochore	✗	[40,43,45–50]
KIF17	✓	not reported		✓	[51]
KIF18B	✗	✓		✓	[52,53]
Lis1	✓	✓	centrosome, kinetochore and cortex	not reported	[54–56]
Nav	✓	not reported		✓	[57]
MCAK	✓	✓	centrosome, kinetochore	✓	[58–60]
Melanophillin	✓	not reported		✓	[61]
MACF	✓	not reported		✓	[62,63]
P140Cap	✓	not reported		✓	[64]
SLAINs	✓	✗		✓	[23]
STIM1	✓	✗	endoplasmic reticulum sheets	✓	[65–67]
TIP150	✓	✓		✓	[68]

Structural aspects of +TIPs and their individual roles in regulating microtubule dynamics have been extensively studied *in vitro* and *in vivo* (reviewed by Akhmanova & Steinmetz [3]), primarily under interphase conditions. In fact, many of the +TIPs that localize to interphase microtubule ends have not yet been tested for their localization in mitosis (table 1). The current model of plus-tip recruitment is largely derived from studies of the interphase cytoskeleton.

### 4.1. Multi-protein complexes at the plus-end: +TIP tracker networks

Autonomous plus-end trackers are +TIPs that allow other +TIPs to be recruited and ‘hitch-hiked’ at the plus-end [20,23,69]. ‘Hitch-hiker’ +TIPs include both microtubule stabilizers and destabilizers. For example, hitch-hiker +TIPs such as CLASPs and CLIPs increase microtubule stability by stimulating pauses and by increasing rescue frequency, respectively [26,70]. Other hitch-hiker +TIPs such as the Kin I family member, MCAK, depolymerize microtubules [58,59,71]. The change in the balance of microtubule stabilizers and destabilizers at the plus-end correlates with microtubule stability, establishing the importance of hitch-hiker +TIPs in defining plus-end dynamics.

+TIPs interact among themselves, and this interaction can alter the function and activity of +TIPs. Two independent examples illustrate this: first, association among +TIPs can release autoinhibitory mechanisms embedded in EB1 and CLIPs [72–75]. CLASPs bind to CLIPs independently from EB1, but both require EB1 for their accumulation onto plus-end and microtubule stabilization [26,27,69,70,76]. Second, interaction between two plus-end-binding proteins (Kif18b and MCAK), which promotes depolymerization, is negatively regulated by Aurora B kinase-mediated phosphorylation of MCAK [52]. Aurora B-mediated phosphorylation of MCAK is a site-specific local event acting on submicrometre scales [60,77]. Thus, through the interactions among +TIPs, a protein network (+TIP tracker network) to finely regulate microtubule dynamics can emerge.

### 4.2. How are autonomous +TIPs loaded at the microtubule ends?

Several tip-tracking mechanisms have been proposed (reviewed by Akhmanova & Steinmetz [3]). Autonomous +TIPs may recognize a feature of microtubules that is physically or chemically different at growing microtubule ends [78–80]. Among autonomous plus-end trackers, two distinct families of proteins are found to directly bind to the plus-ends: (i) tumour over-expressed gene (TOG) domain-bearing proteins [81,82], and (ii) the EB family of proteins [3]. Tandem TOG domains bind tubulin dimers directly and track plus-ends autonomously by a diffusion-facilitated mechanism [82]; however, a generic mechanism to explain the loading of TOG bearing proteins has not yet emerged (reviewed by Al-Bassam & Chang [81]). On the other hand, the EB family of proteins directly bind to plus-ends and track-growing microtubule ends autonomously by recognizing the nucleotide state of the tubulin [76,78,83–85]. In addition, proteins (such as CLIP-170) that are nucleotide-sensitive microtubule-binding proteins are enriched at plus-ends by recognizing both EB1 and tyrosinated α-tubulin [76]. Whether plus-end tracking systems are regulated differently in mitosis to keep up with the increased dynamicity of microtubules or to meet the need of distinct microtubule attachment sites is not known.

### 4.3. How are hitch-hiker +TIPs loaded onto autonomous +TIPs?

EB1 is the most studied of the autonomous +TIPs, and we use it as a prototype autonomous +TIP to discuss hitch-hiker +TIPs. EB1 consists of a calponin homology (CH) domain in the N-terminus and a coiled-coil domain in the C-terminus, which are connected through a flexible linker. The CH domain is required for binding to microtubules. The coiled-coil domain is responsible for protein dimerization, and this in turn contributes to the regulation of microtubule dynamics and loading of other +TIPs [86,87]. So far, two distinctive modes of direct interaction have been identified between EB1 and its interactors, bearing either a cytoskeleton-associated protein glycine-rich (CAP-Gly) domain or Ser-x-Ile-Pro (SxIP) motif.

(1) The CAP-Gly domains are found in CLIPs and dynactin subunit p150. The structural details of interaction between CAP-Gly domain and EB1's EEY/F motif were recently characterized. The CAP-Gly domain contains a highly conserved hydrophobic cavity and unique glycine-bearing motifs that allow the interaction with microtubules or EB1 through its EEY/F motif [74,88,89].
(2) The SxIP motif, a four-amino-acid stretch, found on several +TIPs, binds the EBH domain of EB1. Mutation of IP dipeptides within the motif disrupts EB1 binding and plus-end localization [20,23,69]. Therefore, the SxIP motif has been called a microtubule tip localization signal. However, in the interphase-specific EB1 interactor, SLAIN mutating all of the IP dipeptides did not abolish SLAIN's plus-end localization and EB1 binding [23]. Also, in the mitosis-specific EB1 interactor, Kif18B, the minimal EB1-binding domain is insufficient for Kif18B's efficient binding to plus-end compared with full-length Kif18B [82]. These two findings suggest the presence of additional sequences that contribute to efficient plus-end association. Because EB1 is regarded as a core component of +TIP networks, additional features surrounding the SxIP motif may also be important for discriminating across the various hitch-hiker +TIPs. In strong support of this idea, a recent study has revealed a longer motif (SxIP-9AA) to reliably deduce EB interactors [90]. Better understanding of +TIP interactions will reveal how different hitch-hiker +TIP complexes occupy distinct microtubule subsets.

### 4.4. Are there cell-cycle-specific +TIPs?

Some (but not all) +TIPs are loaded onto plus-ends throughout the cell cycle (table 1). Recent identification of the interphase-specific +TIP SLAIN [23] and mitosis-specific +TIP Kif18b [52,53] is probably only the tip of the iceberg demonstrative of cell-cycle-specific differences in plus-end-bound +TIPs. For instance, whether chTOG1 is loaded on to mitotic plus-ends is unclear.

Additionally, interphase +TIPs that localize to mitotic plus-ends could be regulated through mitosis-specific regulatory events. The autonomous tracker EB1 acts as an anti-catastrophe effector that stimulates microtubule growth [87]; whether EB1 acts similarly in mitosis in all subsets of microtubules is not known.

## 5. Specialized mitotic plus-end structures and their composition

During mitosis, plus-ends anchor dynamically growing and shrinking microtubules to distinct subcellular sites. It is unclear how plus-end fate is differentially regulated at these sites. A starting point could be careful comparison of dynamic changes to the structure and composition of plus-ends at distinct subcellular sites.

### 5.1. Are there distinct spatial domains within plus-ends?

**Figure 2.**
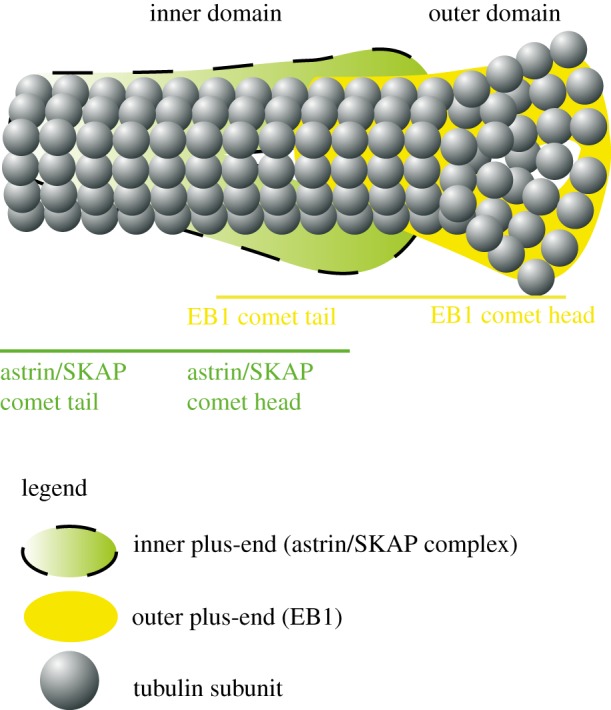
Schematic of inner and outer regions of the microtubule plus-end: the EB1 comet localizes to outer domain of plus-end, whereas the SKAP/Astrin complex associates with the inner domain of the plus-end.

EB1 and EB1-interacting +TIPs, including CLASP1, CLIP170 and dynein–dynactin complex, display a typical comet-like structure. This structure has a bulged head at the outer tip and a tapering tail in the inner side (figure 2). The comets of some +TIPs, such as CLASP1, Astrin and Kinastrin/SKAP, do not fully overlap with the localization of the EB1 comet [91,92] and instead overlap only with the tail of the EB1 comet, suggestive of being prevented from loading onto the outer domain of the comet (figure 2). The term ‘tip trailers’ has been created to describe proteins that occupy the tail region of the EB1 comet [93]. This might be a general feature of EB1 interactors on microtubule ends. The plus-end's spatially distinct outer and inner domains may have domain-specific roles and may be differently regulated throughout the cell cycle.

### 5.2. Comets versus crescents: varying shapes of mitotic +TIP signals

In mitosis, a varying shape of +TIP signal is observed at the kinetochore: EB1 signals appear as crescents, instead of comets [94]. However, this crescent-shaped signal is specific to the growth phase of kinetochore-bound microtubules, confirming EB1's continued association with plus-ends. What causes this change in the shape of EB1 signal? Other additional receptors for EB1 may exist at the kinetochore and change the shape of EB1 signal. Alternatively, the shape change may reflect a unique arrangement or regulation of kinetochore-bound plus-ends. Several lines of evidence support a unique regulation of kinetochore-bound plus-ends. First, like EB1, the signals of Astrin and SKAP at the kinetochore are also not comet-shaped (N. Tamura & V. M. Draviam 2012, unpublished data), suggestive of a general change in signal for kinetochore-bound +TIPs compared with unbound +TIPs. Second, several +TIPs (e.g. Dynein/dynactin complex) normally found at plus-ends are depleted from plus-ends bound to kinetochores [30,48]. Third, the SKA complex that induces the formation of curved microtubules are enriched at kinetochores [95]. Together, they indicate that plus-ends at kinetochores are indeed under special regulation.

## 6. Role of autonomous +TIPs in mitosis

### 6.1. End binding family

As in interphase, EB1 specifically accumulates in the polymerizing end of mitotic microtubules, and it is also targeted to kinetochores and the mitotic cell cortex [94,96,97]. The growing list of evolutionarily conserved EB1 interacting proteins with diverse roles (table 2) and the evidence that EB1 can bind to microtubules autonomously have underscored the importance of EB1 comet platforms that form at the mitotic plus-ends.

**Table 2.** EB1 interactors illustrating +TIP network interactions and function, prepared on the basis of +TIP interaction with at least one another hitch-hiker +TIP. Known EB1 interactors identified from asynchronous cell populations. KT, kinetochore; MT, microtubule; (C), *C. elegans*; (D), *D. melanogaster*; (S.c), *S. cerevisiae*; (S.p), *S. pombe*; (X), *X. laevis.*

EB1 interactors	functions	homologues	interaction with other ‘hitch-hiker’ +TIPs	references
APC	MT stabilization (increasing MT growth and reducing catastrophe), KT-MT attachments, spindle positioning	dAPC1, dAPC2 (D) APR1 (C), (Kar9 (S.c))	MCAK, Dia1	[15–19,37,59, 98–101]
CLASPs	MT stabilization (increasing pause, and restricting catastrophe), KT-MT attachment, Spindle positioning	Orbit/Mast1 (D), CLS-2(C), Stu1 (S.c), Peg1 (S.p)	CLIP170 and CLIP115, MACF1	[26–29,72, 102–104]
CLIPs	MT stabilization (promoting MT rescue), KT-MT attachment	CLIP-190 (D), Bik1 (S.c), Tip1 (S.p)	CLIP115, CLASPs, p150Glued	[27,32,33,70,72]
DDA3	MT depolymerization	not reported	MCAK	[35,36]
Diaphanous (Dia1)	actin polymerization, MT stabilization, cell polarity, migration, golgi architecture, intercellular trafficking of vesicle and organelles	Diaphanous (D), Bni (S.c), Cdc12p (S.p)	CLIP170, APC	[37,38,105–113]
Dynactin (p150)	mediating Dynein interaction with its interactors, MT nucleation, spindle positioning	Glued (D), Dnc-1p(C), Nip100 (S.c), Ssm4p (S.p)	CLIP170	[39,43,45,114,115]
KIF17	MT stabilization, epithelial architecture	OSM-3 (C)	APC	[51]
KIF18B	MT depolymerization, astral MT organization.	not reported	MCAK	[52,53]
MCAK	MT depolymerization	Klp7 (C), XKCM1 (X)	APC, DDA3, TIP150	[35,36,59,116]
SLAINs	MT polymerization	not reported	CLIP170, chTOG1	[23]
TIP150	recruitment of MCAK to MT plus end	ICIS (X)	MCAK	[68]

In mitotic cells, loss of EB1 can disrupt stable positioning of the mitotic spindle and normal alignment of chromosomes, leading to slightly increased incidence of missegregation in anaphase [98,99,117], highlighting EB1's mitotic role in chromosome segregation and spindle positioning. Also, overexpression of the microtubule-binding domain of EB1 perturbs both spindle position and segregation accuracy [99], underscoring the importance of EB1-mediated loading of other +TIPs. While EB1 is clearly essential for proper cell division, loss of EB1 does not yield a severe mitotic defect in spindle assembly or functions as expected from a major plus-end platform, indicative of other plus-end platforms operating during mitosis. These could be other EB family proteins as depletion of EB3 disrupts mitotic progression [118].

### 6.2. Tumour over-expressed gene family

Tandem TOG domains or TOG-like domains are found in chTOG1 and CLASPs (reviewed by Slep [119]). Although the enrichment of CLASP1 at plus-ends is reliant on other +TIPs as well (see §4), chTOG1 can be autonomously recruited and maintained at plus-ends. The *Xenopus* homologue of chTOG1, XMAP215, enriches at plus-ends and acts as a microtubule polymerase as it catalyses the rapid growth of plus-ends [82]. It is not clear whether chTOG1 and EB1 assemble separate sets of +TIP networks, as they both have at least one common interactor, SLAIN [23].

Both CLASPs and chTOG1 are recruited to specific subcellular structures during mitosis. CLASPs are recruited to the outer kinetochore, even in the absence of plus-ends. At the kinetochore, CLASPs promote stability and growth of kinetochore microtubule fibres [28]. chTOG1 is prominently recruited to mitotic centrosomes, and loss of chTOG1 causes a dramatic loss of bipolar spindle assembly and causes the formation of multiple minispindles [120]. It is noteworthy that loss of chTOG1 or CLASPs disrupts mitosis more dramatically compared with loss of EB1 [28,99,121].

### 6.3. Other emerging autonomous plus-end binding proteins

Plus-end-directed kinesins Kif18A and Kif4A, which suppress microtubule dynamics and align chromosomes along the metaphase plate [122,123], are other potential candidates for assembling TIP tracker networks that control cell division. An important future step would be to extract spatially distinct +TIP networks and learn about how they may differentially regulate subsets of mitotic plus-ends.

## 7. Force generation: plus-ends at the cell cortex

Mechanical forces can be generated either by microtubule-bound motors or by harnessing energy associated with plus-end growth and shrinkage. The fundamental concept behind the idea of plus-end-mediated force generation is based on the ‘microtubule as a molecular machine’ model [124]. By anchoring microtubule plus-ends to distinct structures within the cell using selected +TIPs, the plus-end's biochemical transition, defined by GTP hydrolysis, can help generate forces that pull or push against subcellular structures. We first discuss an interphase example to illustrate the complexity involved in cortical microtubule capture. We then discuss the role of a +TIP motor, Dynein, in force generation at the mitotic cell cortex.

### 7.1. Signalling microtubule capture (lessons from interphase cortex)

**Figure 3.**
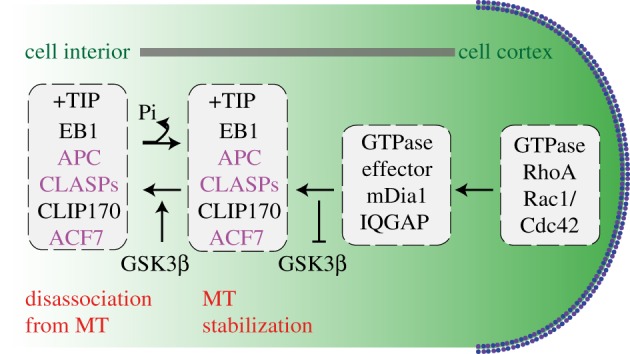
Cartoon of +TIP network that stabilizes microtubules (through a GSK3β lens): +TIP proteins that stabilize microtubules at cell cortex in interphase. In purple are substrates of GSK3β that associate with microtubules in a phosphorylation dependent manner. In dashed boxes are proteins regulated by RhoA or Rac1/Cdc42 GTPases.

The capture and anchoring of microtubule plus-ends to a stable subcellular structure is pivotal for a microtubule-mediated force generation system. A decision to maintain or dissolve microtubule-mediated forces can be made by simply stabilizing or destabilizing the captured microtubule fibre (discussed further in §9). The best illustration of mechanisms by which metazoan +TIPs regulate microtubule capture at the cell cortex emerges from studies of migrating interphase cells where microtubules are stabilized and anchored for minutes (see below and figure 3).

At the actin-rich interphase cortex, microtubule capture is reliant on membrane-bound Rho family GTPases that regulate the association of +TIPs with cortical proteins: the +TIPs, CLIP-170, CLASP2 and adenomatous polyposis coli (APC) interact with an effector of Rac1/Cdc42 GTPases, IQGAP1 (figure 3). IQGAP1 binds to actin filaments in the leading edge of the migrating cell [115,125–127], and thereby act as linker between actin and microtubule cytoskeletons. These interactions are modulated through phosphorylation cascades by GSK3β, a core kinase of several signalling mechanisms (reviewed by Green [128]). GSK3β acts downstream of the Rho GTPase, Cdc42, and phosphorylates CLASP2 and APC [100,115,127]. Loss or gain of function of GSK3β can result in the stabilization or disassociation, respectively, of interactions between CLASP2:IQGAP1, EB1:microtubules and APC:microtubules [91,100,115,127]. Thus, a local signalling cue from the membrane can directly control plus-end dynamics (figure 3).

Another example of a local cue controlling microtubule dynamics is revealed from studies of the actin-binding formin, mDia1 (a RhoA effector). mDia1 is a cortical-binding partner of APC, EB1 or CLIP170, and interaction of mDia1 with APC or EB1 is associated with stable microtubules [37,105]. Through Erb2-mediated Memo-RhoA-mDia1 signalling, mDia1 recruits ACF7, an EB1 interactor, to plasma membrane, and thereby enables microtubule capture at cell cortex [129,130].

In summary, as a first step, extracellular local signals activate the Rho GTPase, Rac1/Cdc42, which results in the recruitment of the appropriate effectors and cortical binding partners of +TIPs and the localized inhibition of downstream kinases such as GSK3β. Subsequently, the accumulated +TIPs may bind and stabilize the microtubule in the vicinity, which can then be captured by the proteins in the leading edge of the cell. Can similar mechanisms operate during mitosis for capturing and anchoring microtubule plus-ends at the cell cortex? CLASP1, EB1, APC and Cdc42 are all essential for proper spindle positioning [99,102,131]. The polarity kinase aPKC required for proper orientation of the spindle in polarized epithelial cells can inhibit GSK3β to locally stabilize microtubules [10]. It is therefore possible that the interphase cortical network of +TIPs and their regulators are deployed again in mitosis to regulate astral microtubule capture, keeping in mind that the rates of microtubule turnover are considerably different during migration and mitosis.

### 7.2. Dynein as a plus-end-tethering and cortical microtubule-pulling force

Microtubule cortex interaction mediated through Dynein/dynactin motor protein complex is better understood in yeast and worm models [132–135], wherein the minus-end-directed Dynein motor is able to generate microtubule pulling forces, which in turn rotates the spindle. Support for Dynein's role in single-handedly tethering onto plus-ends and generating microtubule pulling forces were demonstrated *in vitro* [136,137]. *In vivo* support for a plus-end-tethering and microtubule-end-pulling role of Dynein exists in yeasts [138], but not in other systems. In human cells, microtubule lattice-bound protein MAP4 that interacts with Dynein/dynactin complex is able to inhibit Dynein-mediated force generation and perturb spindle movement, without altering plus-end dynamics [102]. These slightly conflicting findings could arise from differences in model systems wherein the extent to which Dynein along microtubule walls and Dynein at microtubule ends contribute to the pulling forces may vary. It is well known that Dynein/dynactin localizes all along the microtubule wall, as well as the plus-end in mammalian cells [39]. Therefore, to understand mechanisms that control Dynein-mediated pulling, it would be important to identify cortical receptors of Dynein/dynactin complex (see below).

NUMA and LGN complexes that remain on the cell cortex and disassociate from Dynein complex in a PlK1-dependent manner [139] are good candidates for being direct interactors and cortical receptors for Dynein. NUMA binds directly to microtubules and indirectly to Galphai [140], a G-protein that destabilizes microtubules *in vitro* [141,142]. Other candidates for being cortical receptors of Dynein during mitosis include the interphase-cortex-bound regulators of Dynein: LIS1, a Dynein interactor localizing to the leading edge of cells [54,114], and dynactin complex p62 subunit recruited to the interphase cortical region [143]. Regulation through cortex-bound LIS1 and p62 are particularly important options as they can directly influence Dynein's motor activity at cell cortex, without perturbing Dynein's localization *per se*.

Although Dynein can single-handedly carry out the chemical function of tethering and pulling microtubules, the biological function of Dynein is likely to be tuned through more than one regulator to selectively stabilize correct microtubule interactions and also to coordinate pulling forces, all along the microtubule wall or among the various microtubule ends, while the spindle is being guided towards a predetermined position. Evidence for multiple independent ways for microtubule interaction with the cortex-bound Dynein/dynactin subunits exists in interphase: either directly, through EB1 or through CLIP170 [72]. To what extent Dynein's various microtubule interactions contribute towards Dynein's function in spindle orientation in mitosis remains to be explored.

## 8. Plus-end regulation at chromosome–microtubule attachment sites

Proteins of the kinetochore to which plus-ends attach and impart forces to power chromosome movement have been biochemically characterized and their roles in chromosome segregation described (reviewed by Cheeseman & Desai [9]) [95,144,145]. At the kinetochores, at least two distinct kinds of plus-end regulation has been observed. (i) Kinetochore proteins that interact with microtubules such as Ndc80/HEC1 or CENP-E [146–148] become associated close to microtubule ends at kinetochores and may serve as ‘tethers’ to maintain stable microtubule attachment. They ensure the time period and physical orientation of kinetochore–microtubule interaction. (ii) Outer kinetochore localizing +TIPs such as MCAK and Ska1 complex regulate kinetochore-fibre dynamicity [60,95,149–151]. These serve as ‘governors’ of microtubule dynamics as they directly control tubulin subunit addition/removal. Recent work shows that Ndc80/HEC1 is also able to bind to plus-ends and regulate plus-end dynamics *in vitro* [152], suggesting both a ‘tether’ and ‘governor’ role for this kinetochore protein.

+TIPs in general are important not for the congression of all chromosomes but only a subset [92,99,153]. Whether this is due to redundancy in congression pathways or due to random positions taken by the chromosomes within the spindle is not fully understood.

+TIP localization at the kinetochore is finely regulated. Some +TIPs such as Astrin/SKAP complex and EB1 are delivered by plus-ends to the kinetochore only after microtubule attachment, and they play distinct roles in chromosome congression [92,94,99,153]. These +TIPs are in a good position to act as both ‘tether’ of plus-ends and as ‘governor’ of plus-end dynamics. On the other hand, +TIPs such as Lis1, CLIP-170 and Dynein–dynactin complex are present at the kinetochores only in the absence of microtubules. In fact, constitutively retaining the kinetochore localization of +TIPs that are normally lost from kinetochores following kinetochore–microtubule attachment has deleterious consequences: a Zwint mutant that fails to release RZZ complex from the kinetochore stably retains Dynein complex at kinetochores and arrests cells in mitosis [154]. This evidence underscores the importance of maintaining a specialized state of plus-end-associated protein composition at the kinetochore.

Phosphorylation–dephosphorylation cycles of distinct +TIPs at kinetochores, in an Aurora-B kinase-dependent manner, can promote either microtubule assembly or disassembly [77,155–157], highlighting the role of kinases in controlling microtubule dynamics through +TIPs. The growing list of EB1 interactors that are modified through phosphorylation reveals a range of phosphorylation changes that have been visualized *in vivo* (table 3). This is an interesting point to note, as there is no example, so far, of kinases controlling plus-end loading of +TIPs *in vitro*, although *in vivo* evidence exists. Whether the kinases specifically act on plus-end-bound +TIPs or on +TIPs in the local environment will become apparent with *in vitro* studies.

**Table 3.** Phosphorylation-mediated regulation of +TIP localization and function. Phosphorylation sites on interactors of EB1, upstream kinases and the role of phosphorylation in regulating microtubule function are all tabulated. Note that localization of +TIPs to distinct subcellular sites is modulated in a phosphorylation-dependent manner. In blue and red are sites phosphorylated specifically in mitosis and interphase, respectively.

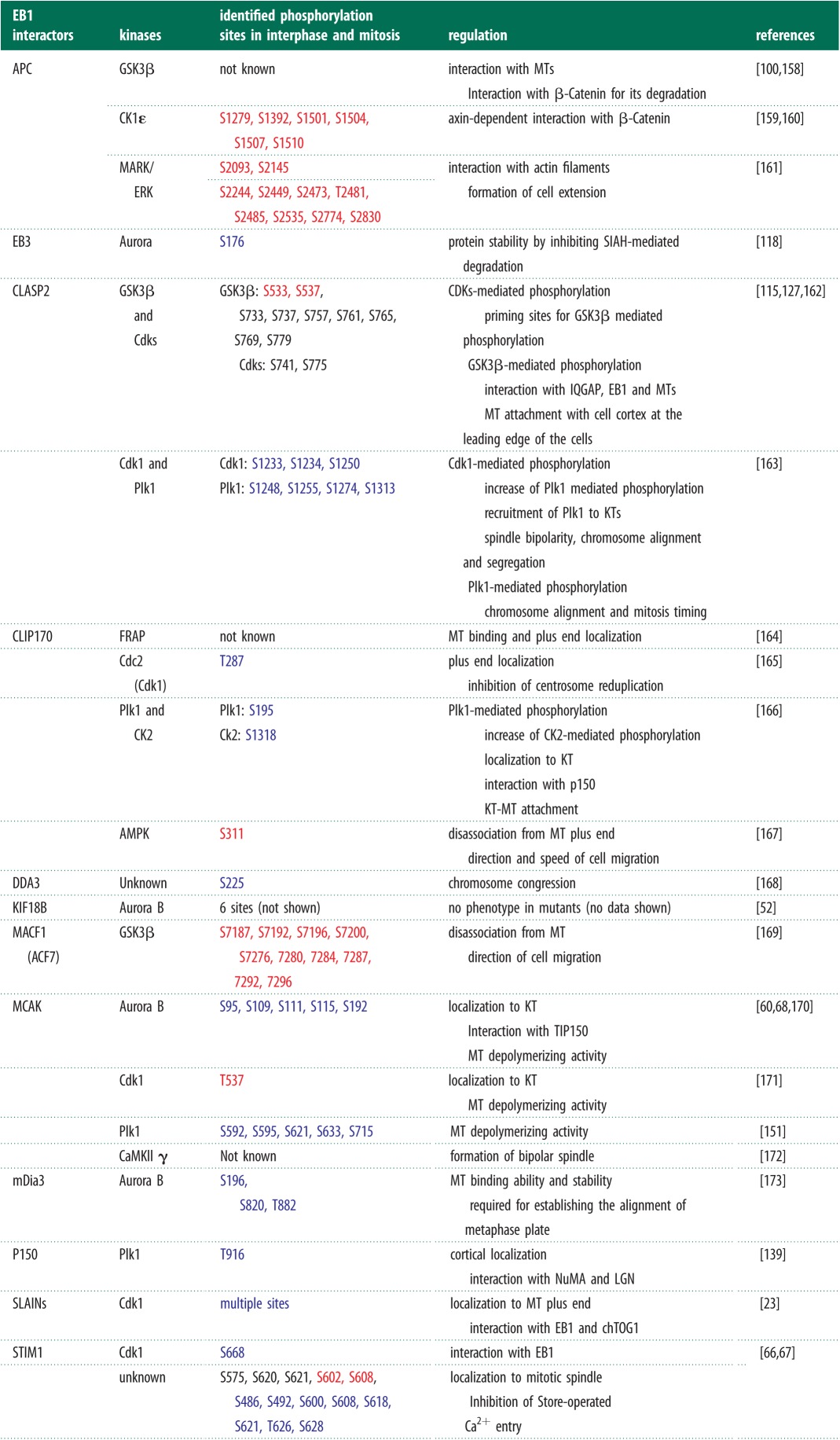

## 9. Exciting possibilities: can microtubule plus-ends act as ‘mobile platforms’ that distinguish incorrect versus correct states of microtubule capture?

**Figure 4.**
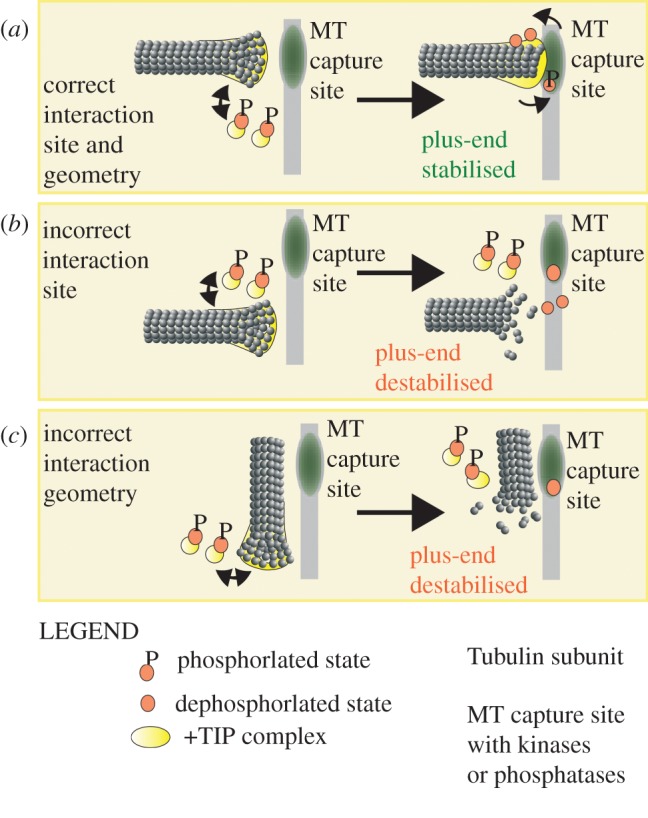
Speculative model of plus-end-bound +TIPs as ‘mobile platforms’ that ensure microtubule capture at correct subcellular sites and in proper geometry. In this model, +TIPs within a mitotic cell are predominantly maintained in a phosphorylated state that reduces +TIPs' affinity for microtubules. A limited pool of non-phosphorylated +TIPs is present and they load onto mitotic plus-ends but are rapidly lost from them owing to mitosis-specific phosphorylation. However, when the ‘mobile platform’ is brought into the vicinity of a microtubule capture site, it is exposed to a dephosphorylation-based signalling cascade that extends +TIP lifetime by counteracting phosphorylation. Thus, the dephosphorylation-based signalling cascade stabilizes the plus-end interaction. (*a*) Plus-end interaction at correct microtubule capture site and in an end-on geometry. Phospho-changes to proteins on the plus-end prevent microtubule disassembly. (*b*,*c*) Plus-end interaction (*b*) away from the microtubule capture site or (*c*) in an improper lateral geometry. Microtubule end stabilizing dephosphorylation of proteins on the plus-end fails to occur. No microtubule stabilizing signals reach from the interaction site, leading to rapid disassembly of the microtubule end.

The precise biochemical nature of the signal that mitotic cells use to distinguish between incorrect versus correct microtubule capture is not known. We propose a model in which the plus-end together with +TIPs serve as a ‘mobile platform’ that receives a signal from plus-end interaction sites to either destabilize or stabilize microtubules. In this model, an enzyme at the plus-end's interaction site modifies the ‘mobile platform’, and thereby generates a biochemical signal that stabilizes microtubules in a self-instructive manner. In the absence of a correct plus-end interaction, the unmodified ‘mobile platform’ would simply disassemble and destabilize the plus-end. Through such a signalling process, cells can ensure (i) microtubule capture at correct subcellular sites, and (ii) microtubule interaction in correct geometry (figure 4).

The ‘mobile platform’ model gains support from at least three distinct observations. (i) Mitotic plus-ends load and unload +TIP complexes dynamically, and therefore are ideal sensors for dynamically responding to incorrect *versus* correct microtubule capture. (ii) Phosphorylation-mediated changes in +TIPs often decrease their affinity for microtubules and destabilizes microtubules (table 3) [52]. If correct microtubule interactions were to expose +TIPs to phosphatases at the microtubule capture site then phosphorylation state changes to +TIPs could extend the +TIPs plus-end residence time and stabilize the microtubule capture event. Conversely, incorrect microtubule interactions that fail to expose the +TIPs to phosphatases will fail to be stabilized. (iii) Because several +TIPs are kinases [90] and some +TIPs such as CENP-E can deliver PP1 [148], a signalling cascade may also be locally amplified through enzymes at both the plus-end and microtubule capture site.

In this model, we generalize that +TIPs that favour microtubule capture exist predominantly in a phosphorylated state in mitosis. Hence, the phosphatase's role would simply be to relieve the phosphorylation and lengthen +TIP residence on microtubule ends. In fact, the B56-PP2A phosphatase is required for stable kinetochore–microtubule interactions [174]. Whether phosphatases are required to lengthen the +TIP lifetime could easily be tested by photobleach studies of +TIPs in distinct subsets of microtubules.

Evidence for site-specific, phosphorylation-based release of incorrect microtubule interaction exists between centromeric Aurora kinase and its substrates at the inner and outer kinetochore [60,77]. These examples address the establishment or release of a previously stable microtubule attachment, and not the initial microtubule capture *per se.* By contrast, the ‘mobile platform’ model looks at plus-ends receiving or delivering signals for differentially stabilizing correct versus incorrect microtubule capture. The ‘mobile platform’ model has at least two distinguishing features. (i) The model allows quick response. Correct plus-end interactions, in an end-on fashion, that deliver +TIPs to microtubule interaction sites will alone be stabilized. (ii) The model can scale up. The large number of plus-ends (in the order of thousands in mammalian cells), searching the entire volume of cell to identify submicrometre-sized microtubule interaction sites, mean that majority of plus-ends would make incorrect or no interactions with microtubule capture sites. Hence, the system should disassemble incorrect interactions, which is possible within the self-instructive ‘mobile platform’ model. Thus, the ‘mobile platform’ model allows for a highly sensitive signalling system that can selectively stabilize correct interactions and robustly destabilize incorrect interactions.

Our model of plus-ends as ‘mobile-platforms’ that distinguish incorrect versus correct attachments predicts that different +TIP complexes loaded at distinct subcellular sites of microtubule capture (table 2) would play a key error correction role. Therefore, it is worth investigating the extent to which the loading of a subset of +TIP complex may be necessary to distinguish between incorrect versus correct microtubule capture at distinct microtubule capture sites.

Determining the mechanisms that regulate the loading and unloading of +TIPs at different microtubule interaction sites may reveal signalling roles of plus-ends in distinct microtubule-mediated mitotic events. In this context, considering mitotic plus-ends as ‘moving platforms’ with distinct cytoskeletal anchoring, biochemical signalling and force-coupling roles is likely to provide a clear framework for elucidating the impressive cellular feat of rapidly moving bulky subcellular structures.

## 10. Acknowledgements

We thank Draviam group members and Marisa Segal for comments on the review and stimulating discussions. This work is supported by grants to V.M.D. from Cancer Research UK and Royal Society, UK.
